# 

**Published:** 1891-05

**Authors:** 


					﻿MAT1 1891'
OLD SERIES
Vol. XXV
. & 1855 6 *
4a
B JOIIRNAESE

, H. V. M. MILLER, M. D., LL.D. J
VIRGIL 0. HARDON, M. D.
F. W. McRAE, M. D.
L.	P. KENNEDY, A. M... M. D. /
M.	B. HUTCHINS, M. D
L. B. GRANDY, M. D.
MrUSHED By JAMES P.HARRISON&C^
ME______________«flTLANTA,CA. M
SUBSCRIPTION I 12.00 A YEAR.
i ut Afinnfx. . Aft HRf'oixl■ r-lsisR tn.ittPT.
				

